# Synergistic Removal of Pb(II), Cd(II) and Humic Acid by Fe_3_O_4_@Mesoporous Silica-Graphene Oxide Composites

**DOI:** 10.1371/journal.pone.0065634

**Published:** 2013-06-11

**Authors:** Yilong Wang, Song Liang, Bingdi Chen, Fangfang Guo, Shuili Yu, Yulin Tang

**Affiliations:** 1 The Institute for Biomedical Engineering and Nano Science, Tongji University School of Medicine, Shanghai, P. R. China; 2 State Key Laboratory of Pollution Control and Resource Reuse, College of Environmental Science and Engineering, Tongji University, Shanghai, P. R. China; University of Kansas, United States of America

## Abstract

The synergistic adsorption of heavy metal ions and humic acid can be very challenging. This is largely because of their competitive adsorption onto most adsorbent materials. Hierarchically structured composites containing polyethylenimine-modified magnetic mesoporous silica and graphene oxide (MMSP-GO) were here prepared to address this. Magnetic mesoporous silica microspheres were synthesized and functionalized with PEI molecules, providing many amine groups for chemical conjugation with the carboxyl groups on GO sheets and enhanced the affinity between the pollutants and the mesoporous silica. The features of the composites were characterized using TEM, SEM, TGA, DLS, and VSM measurements. Series adsorption results proved that this system was suitable for simultaneous and efficient removal of heavy metal ions and humic acid using MMSP-GO composites as adsorbents. The maximum adsorption capacities of MMSP-GO for Pb(II) and Cd (II) were 333 and 167 mg g^−1^ caculated by Langmuir model, respectively. HA enhances adsorption of heavy metals by MMSP-GO composites due to their interactions in aqueous solutions. The underlying mechanism of synergistic adsorption of heavy metal ions and humic acid were discussed. MMSP-GO composites have shown promise for use as adsorbents in the simultaneous removal of heavy metals and humic acid in wastewater treatment processes.

## Introduction

Heavy metal ions and humic acid (HA) in underground water pose a severe threat to public health and ecological systems.[1]–[3] A great deal of effort has been made to develop effective adsorbents with different chemical compositions, microstructures, and surface functionalities for the removal of heavy metal ions and organic pollutants from water.[4]–[9] In this way, the development of adsorbents with high adsorption capacity, low toxicity, and efficient separation has attracted considerable interest. [10], [11].

In the past decades, silica-based mesoporous materials, which are robust inorganic solids, have shown good potential in water treatment applications due to their large specific surface area (200–1500 m^2^ g^−1^) and accessible surface functionalization. [12], [13] Mesoporous silica groups containing amino and thiol groups have been extensively used to accumulate the metal ions from aqueous solutions.[14]–[20] Recently, magnetic nanoparticles have been introduced in mesoporous silica adsorbents, contributing to direct enhancement of the adsorption of metal ions and possible recovery by magnetic separation [21]–[23].

Recently, the development of bifunctionalized adsorbents capable of enabling simultaneous removal of two kinds of environmental pollutants has become an emerging frontier in the field of water treatment. [24] However, synergistic adsorption of heavy metal ions and humic acid molecules is still a challenge because of their competitive adsorption onto most adsorbents.[25]–[27] To the best of our knowledge, there have been few studies that have evaluated the use of functionalized magnetic mesoporous silica and graphene oxide in the synergistic adsorption of two kind of pollutants. The high surface-to-volume ratio and surface functionalities of the graphene oxide (GO) and PEI-grafted magnetic mesoporous silica (MMSP) microspheres can be both used to this effect. The addition of GO can further improve the colloidal stability of the MMSP.

Herein, we report a facile method for preparation of magnetic mesoporous silica-graphene oxide (MMSP-GO) composites and describe our investigation of the synergistic adsorption of HA and heavy metal ions, specifically Pb(II) and Cd(II). First, the magnetic mesoporous silica microspheres were synthesized and functionalized with PEI molecules, followed by chemical conjugation with the carboxyl groups on GO sheets. The functionalization of the mesoporous silica microspheres containing PEI molecules was found to both facilitate the adsorption of HA and provide sites for chemical reaction with graphene oxide sheets. The adsorption isotherm of Pb(II) and Cd(II) by MMSP-GO composites were studied. And the underlying mechanism of synergistic adsorption of heavy metal ions and humic acid were discussed. The magnetic cores of MMSP microspheres were also embedded to facilitate easy separation using magnetic fields.

## Experimental procedures

### 2.1. Materials

Pb(NO_3_)_2_, CdCl_2_·2.5H_2_O, iron (III) chloride hydrate (FeCl_3_·6H_2_O), sodium acetate, ethylene glycol, ammonium hydroxide (NH_4_OH, 28 wt%), and hydrochloric acid (37 wt% aqueous solution) were purchased from Shanghai Reagent Company (China). Tetraethoxysilane was purchased from Sigma-Aldrich (U.S.). Hexadecyltrimethoxysilane (C_16_TMS) and polyethyleneimide (PEI, 99%, Mw = 1800) were purchased from Alfa Aesar (U.S.). Humic acid was purchased from Aldrich Chemical Company (China) and treated using the method described in our previous experiment. [28] Graphene oxide was purchased from Nanjing XF Nano Company (China). All these reagents were used without further purification. The deionized (DI) water used for the preparation of reagents was purified by Millipore reverse osmosis (RO).

### 2.2. Instruments

The TEM samples were dispersed in DI water and dried onto carbon-coated copper grids before examination. Transmission electron microscope (TEM) images were obtained with a Philips Tecnai 20 transmission electron microscope. Scanning electron microscope (SEM) images were taken using a JEOL SM4800 scanning electron microscope. FTIR spectra were obtained with a Bruker Tensor 27. The TGA data was obtained with a thermogravimetric analyzer (NETZSCH TG209) conducted under nitrogen atmosphere from ambient to 1173 K with the rate of heating at 283 K min^−1^. The magnetic properties were measured at room temperature using a Vibrating Sample Magnetometer (VSM 7407, Lake Shore, USA). The surface zeta potentials of the microspheres were measured using a DLS Particle Size analyzer (Zetasizer Nano-ZS, Malvern, UK.).

The concentrations of total Cd (II) and Pb (II) were determined by an ICP-AES (ICP-optima 2001DV, Perkin-Elmer, USA). Humic acid was determined using a UV-vis spectrophotometer at 254 nm wavelength. The pH of the solutions was measured using a pH meter (pHS-3C model, Leici, China).

### 2.3. Preparation of MMSP-GO Composites

#### 2.3.1. Synthesis of PEI-modified magnetic mesoporous silica microspheres

*Synthesis of Fe_3_O_4_ microspheres*. The magnetic microspheres were prepared using a solvothermal reaction. [29] Then 2.16 g of sodium acetate and 0.86 g of FeCl_3_·6H_2_O were dissolved in 30 mL of ethylene glycol under vigorous magnetic stirring. The homogeneous yellow solution was transferred to the Teflon-lined stainless-steel autoclave and heated to 200°C for nearly 8 h. After that, the autoclave was cooled to room temperature. The products were washed three times with ethanol and DI water and redispersed into DI water for use.

*Synthesis of core-shell structure magnetic mesoporous silica composite microspheres.* First, 4 mL of Fe_3_O_4_ aqueous dispersion (about 100 mg mL^−1^) was treated with 0.1 M HCl aqueous solution under sonication for 20 min. The treated Fe_3_O_4_ microspheres were dispersed in the mixture of ethanol and water (v/v = 70/30) for a while. Ammonium hydroxide was added to adjust pH value of the dispersion to about 9.5. Then 0.12 mL of TEOS was poured into the reactor with vigorous stirring at room temperature. The reaction lasted for about 24 h. The extra reactants were washed four times with ethanol and removed from Fe_3_O_4_@SiO2 microspheres. Then 150 mL of the mixture of ethanol and DI water (v/v = 88/12) was treated with 7.2 mL of ammonium hydroxide. Then 5 mL of ethanol solution of TEOS and C_16_TMS (molar ratio of 4.7∶1) was dropped into the reaction system at the rate of one drop every 10 seconds. The mixture was stirred at constant temperature for 6 h and then the reaction was stopped. The product was washed twice with ethanol and DI water and then calcinated at 550°C for 6 h, producing magnetic mesoporous silica composite microspheres.

*Surface modification of magnetic mesoporous silica composite microspheres with PEI molecules.* In 35 mL of DI water, 20 mg of as-synthesized magnetic mesoporous silica microspheres was dispersed under sonication. Then 30 mg of PEI (Fw = 1800) was added to the aqueous dispersion with vigorous mechanical stirring and sonication. After 30 min, the resulting MMSP were washed three times with DI water.

#### 2.3.2. Synthesis of MMSP-GO composites

In a 50 mL centrifuge tube, 20 mg of GO sheets were dispersed in 30 mL of DI water with 1 h sonication. Into this yellow-brown homogeneous dispersion, 0.080 mL of newly-produced EDC aqueous solution at a concentration of 4.0 mg mL^−1^ was added. This mixture was vortexed for 8 min. Then 2 mg of PEI functionalized magnetic mesoporous silica microspheres were introduced. The reaction lasted for 2.5 h in an end-over-end reactor and the final products were purified by washing three times with DI water, producing MMSP-GO composites.

### 2.4. Adsorption of Pb(II)/Cd(II) and HA

Batch experiments were employed to evaluate Pb(II) and Cd(II) or HA adsorption characteristics under various conditions. All the adsorption experiments were conducted at room temperature (25±1°C). The average values of triplicate measurements were reported and all standard errors were smaller than 5%.

The batch adsorption procedure consisted of (a) distributing 1.0 mg of MMSP-GO or MMSP through 10 mL water solution containing selected concentrations of Pb(II) and Cd(II) in a series of 20 mL glass tubes; (b) adjusting the pH to 2.0–9.0 using 0.1 M stock of HNO_3_ and NaOH solution; (c) adding HA stock solution to each of the tubes to specific preselected concentrations; (d) sealing and shaking all tubes in an incubator at 150 rpm; (e) separating the suspension using an external magnetic field; (f) detection of the residual Pb(II) and Cd(II) concentrations with ICP-AES and detection of the residual concentration of HA with an UV-vis spectrophotometer; (g) calculation of the amounts of heavy metal and HA adsorbed using Eq. (1):
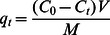
(1)


Here *q_t_* (mg/g) is the amount of heavy metal or HA adsorbed at time t, *C_0_* (mg L^−1^) is the initial heavy metal or HA concentration. *C_t_* (mg L^−1^) is the heavy metal or HA concentration at time t. *V* (mL) is the volume of heavy metal or HA solution, and *M* (g) is the adsorbent mass.

## Results and Discussion

### 3.1. Material Characterizations

**Figure 1:**
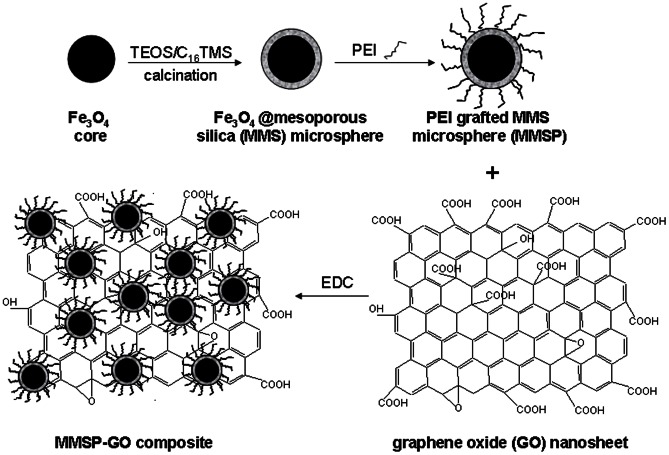
Scheme of the preparation pathway. Schemetic illustration of pathway for preparation of polyethylenimine-modified magnetic mesoporous silica and graphene oxide (MMSP-GO) composites.

The typical synthesis of the MMSP-GO composites is shown schematically in Figure 1. Fe_3_O_4_@mesoporous silica (MMS) core-shell microspheres were first prepared using standard methods of mesoporous silica shell formation via a sol-gel process based on the magnetic microspheres obtained through a solvothermal reaction. [29] C_16_TMS, a coupling agent, was used as the porogen during the silica coating on the magnetic cores, which could be easily removed by calcination. Then monodispersed MMS core-shell microspheres with regular mesoporous silica shells were produced. In order to connect the magnetic mesoporous composites to the graphene oxide sheets, PEI branched molecules were used to modify the surface of the mesoporous microspheres by electrostatic adsorption. [30] The dramatically change in the surface potential of the mesoporous microspheres from −15 mV to +37 mV under the neutral conditions proved that the modification was successful. Next, the formation of MMSP-GO composites was carried out by chemical reaction of MMSP and original GO nanosheets in the presence of 1-ethyl-3-(3-dimethyaminopropyl) carbodiimide (EDC) (Figure 1). In this case, PEI molecules played bilateral roles one of them serving as the binding bridge for conjugation and the other to improve the adsorption affinity of the MMSP microspheres to the pollutant matter. [28].

**Figure 2:**
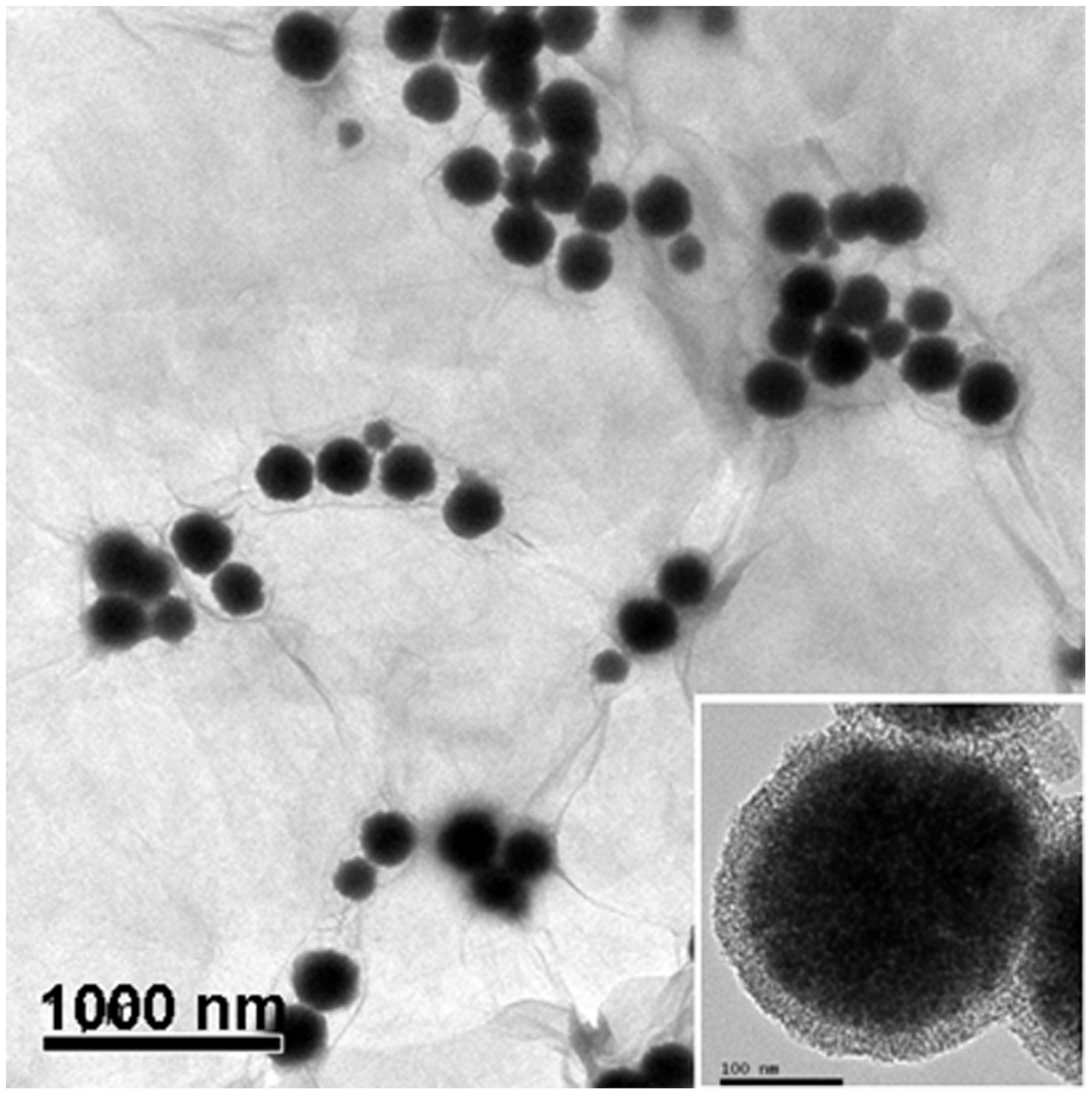
TEM images of the composites. TEM image of polyethylenimine-modified magnetic mesoporous silica and graphene oxide (MMSP-GO) composites (inset is the enlarged TEM image of an individual Fe_3_O_4_@mesoporous silica microsphere on the GO sheet).

Figure 2 shows a TEM image of MMSP-GO composites. The monodispersed MMSP microspheres were distributed on the GO surface. Some microspheres adhered to each other, leaving most of the GO surface exposed. There was little aggregation or multilayer accumulation of the MMSP microspheres, implying good connections between the microspheres and the GO sheets. The average diameter of the MMSP microspheres was 260 nm. The microstructures of the MMSP microspheres were shown in the inset of Figure 2. There was an obvious mesoporous silica shell on the Fe_3_O_4_ core. The loading density of MMSP microspheres on the GO surface could be well controlled maintaining the balance between strong adsorption properties and magnetic resonance.

**Figure 3:**
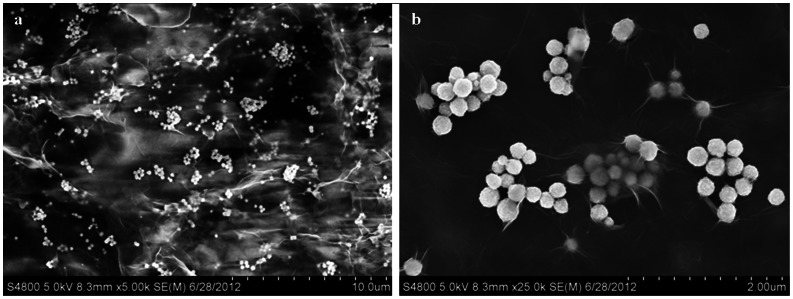
SEM images of the composites. SEM images of polyethylenimine-modified magnetic mesoporous silica and graphene oxide (MMSP-GO) composites.

Figure 3 shows SEM images of MMSP-GO composites at different magnifications. In Figure 3a, the low-magnification SEM image indicates that the MMSP microspheres were distributed homogeneously across the whole surface of the GO. As shown, the MMSP microspheres were firmly anchored on both sides of the wrinkled GO nanosheets (Figure 3b). These GO layers may play a critical role in preventing MMSP microspheres from aggregating in solution.

**Figure 4:**
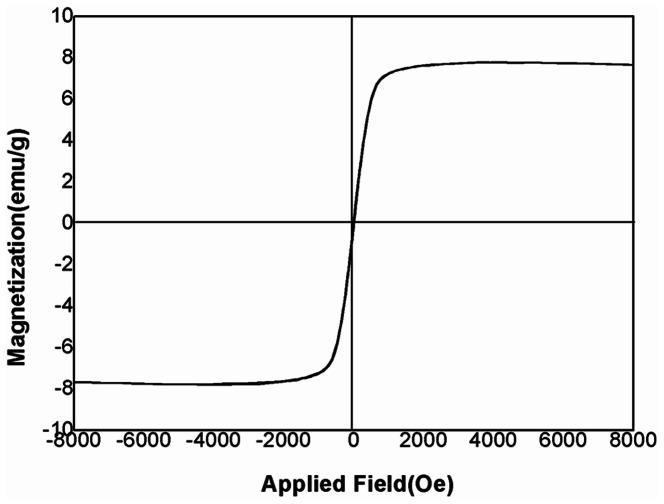
Magnetic property of the composites. Magnetic property of the polyethylenimine-modified magnetic mesoporous silica and graphene oxide (MMSP-GO) composites.

Figure 4 gives magnetic hysteresis loops for MMSP-GO composites measured at 300 K. The profile of the magnetization curve of the MMSP-GO composites showed their typical superparamagnetic properties. The saturation magnetization of the MMSP-GO was 7.5 emu g^−1^, which ensured effective magnetic separation after adsorption equilibrium.

**Figure 5:**
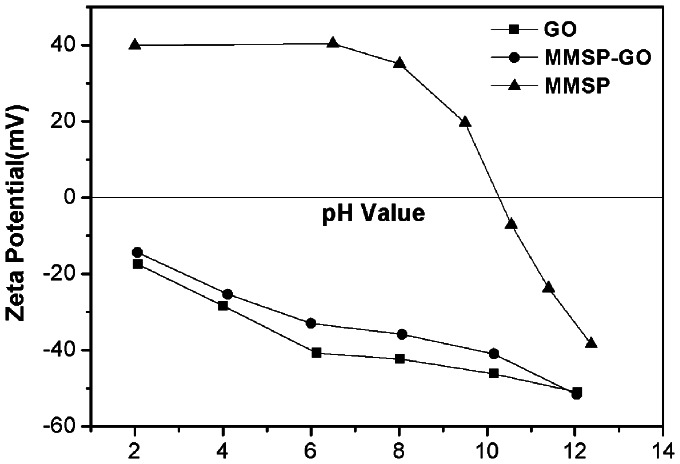
pH-zeta potential relation curves. pH-zeta potential relation curves of polyethylenimine-modified magnetic mesoporous silica and graphene oxide (MMSP-GO) composites, MMSP microspheres, and GO nanosheets, respectively.

Zeta potentials of MMSP-GO composites and corresponding two components of GO and MMSP as a function of solution pH are shown in Figure 5. The point of zero charge (pH_pzc_) of MMSP was 10.4 because of the existence of large numbers of PEI molecules. Points of zero charge (pH_pzc_) of MMSP-GO composites and GO nanosheets approached 1.0. This was because both of them had many oxygen-containing groups on their surfaces. This showed that a majority of the functional groups of GO were still present after the formation of the composites.

**Figure 6:**
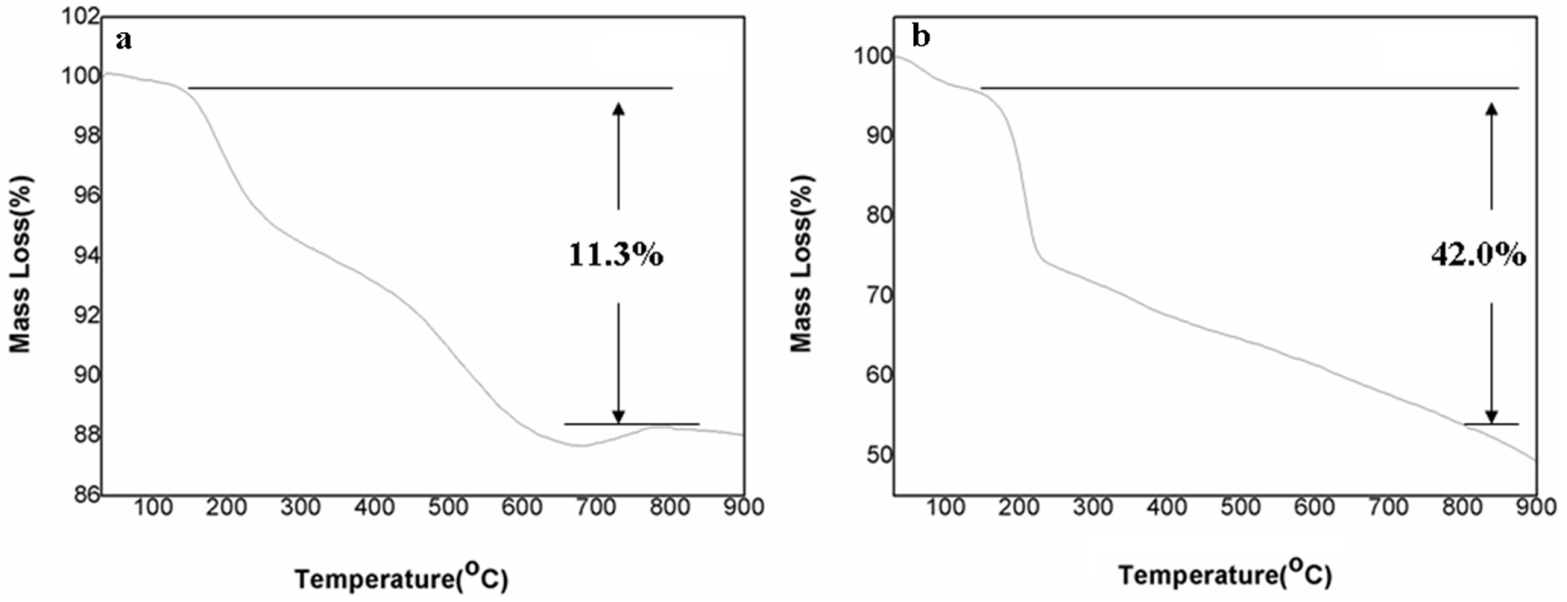
TG analysis curves. TG analysis of (a) polyethylenimine-modified magnetic mesoporous silica and graphene oxide (MMSP-GO) composites and (b) polyethylenimine-modified magnetic mesoporous silica microspheres.

To calculate the composition partition of microspheres to GO nanosheets, we performed TGA analysis of the MMSP-GO composites and MMSP microspheres (Figure 6). The big mass loss of the composites (42.0%) at temperature between 150 to 800°C was due to removal of oxygen-containing functional groups of GO and PEI molecules on MSP microspheres. The loss of mass of MMSP microspheres within a given temperature range was about 11.3% due to the removal of PEI molecules. The weight loss of GO sheets in this range due to the groups of COOH, OH, C = O, and epoxide was about 44.5%. [27] The combined analysis of these TGA results of the composites with each component indicated that the mass partition of the MMSP microspheres was about 7.5%, which was consistent with the reaction stoichiometry.

### 3.2. Investigation of Adsorption of Pb(II)/Cd(II) by MMSP-GO or MMSP

**Figure 7:**
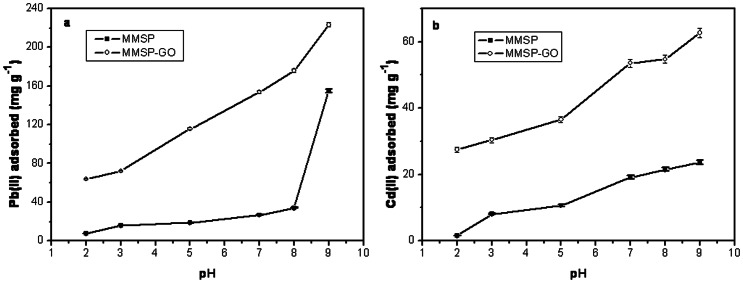
Comparison of adsorptive capacities of heavy metal ions by different materials. a. Effects of pH on adsorption of Pb(II) by polyethylenimine-modified magnetic mesoporous silica (MMSP) and polyethylenimine-modified magnetic mesoporous silica and graphene oxide (MMSP-GO) (Initial concentrations: 20 mg L^−1^; adsorbent loading: 100 mg L^−1^); b. Effects of pH on adsorption of Cd(II) by MMSP and MMSP-GO (Initial concentrations: 20 mg L^−1^; adsorbent loading: 100 mg L^−1^).

We performed batch adsorption experiments with Pb(II) and Cd(II) using MMSP-GO composites and MMSP microspheres. The adsorption capacities of Pb(II) and Cd(II) both increased as pH value increased, peaking at pH 9.0. The same trend was observed for each heavy metal ion. The adsorptive property of MMSP-GO composites was better than that of MMSP microspheres. This was because the GO nanosheets had a stronger absorptive ability than MMSP microspheres (Figure S1). For Pb(II) removal, the maximum adsorption at an equilibrium between MMSP-GO composites and MMSP microspheres was 220 and 152 mg g^−1^, respectively, at an initial metal ion concentration of 20 mg L^−1^. For Cd(II) removal, the maximum adsorption at equilibrium of MMSP-GO composites and MMSP microspheres was 65 and 23 mg g^−1^, respectively (Figure 7). All results indicated that the composites containing GO were able to adsorb more heavy metal ions than those without GO. By comparison, the adsorptive capacity of Pb(II) was higher than that of Cd(II), which resulted from the increased utilization of amino groups on MMSP. [31] At the same time, GO has a greater saturation adsorptive capacity for Pb(II) than Cd(II). [32] It was reported that electrostatic interaction maybe the predominant driven force for adsorption of heavy metal ions by GO materials. [27] In this case, majority of Pb(II) and Cd(II) were adsorbed by GO sheets due to the electrostatic interaction between the GO with negative surface charges and cations in the broad pH range.

### 3.3. Adsorption Isotherms

**Figure 8:**
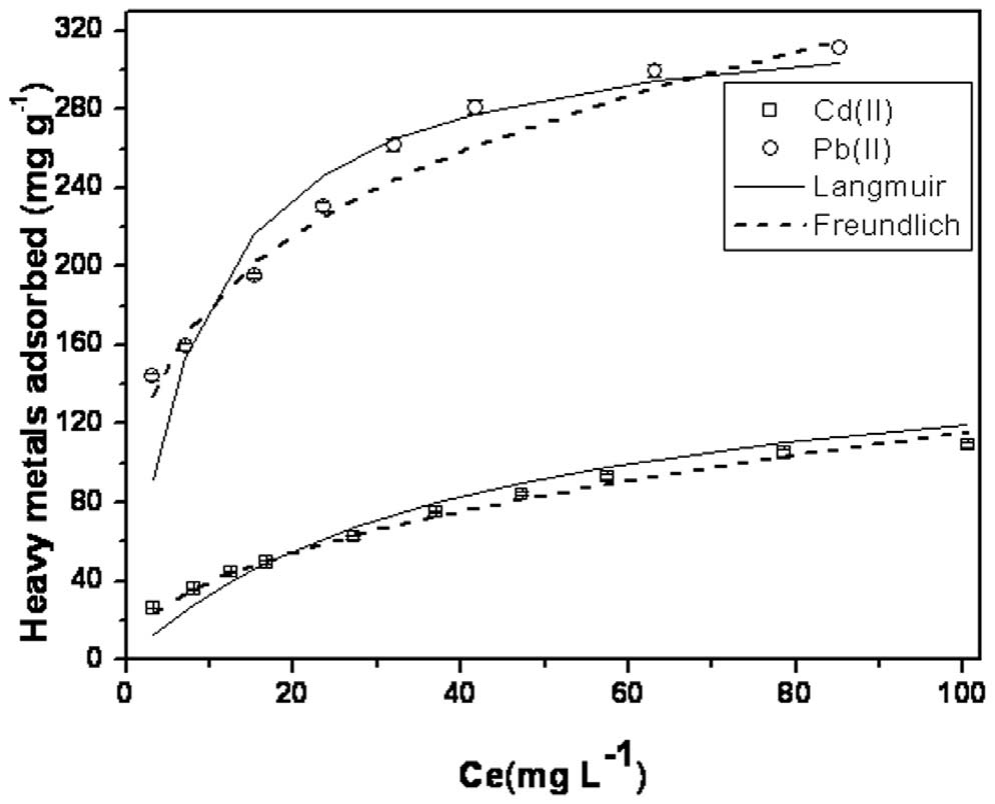
Pb(II) and Cd(II) adsorption isotherms on MMSP-GO. Pb(II) and Cd(II) adsorption isotherms on MMSP-GO (adsorbent loading: 100 mg L^−1^; pH: 7.1; contact time: 24 h ). The solid lines are Langmuir model simulation, and the dotted lines are Freundlich model simulation.

Adsorption isotherm is important for determining the adsorption behavior of an adsorbent. Pb(II) and Cd(II) adsorption isotherm of MMSP-GO were investigated at pH 7.1. The maximum Pb(II) and Cd(II) adsorption amounts on MMSP-GO within the tested concentration range in Figure 8. The equilibrium data were fitted by Langmuir and Freundlich model.
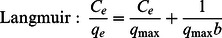
(2)

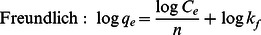
(3)where, *q_max_* (mg g^−1^) is the theoretical maximum heavy metals adsorption amounts, *q_e_* (mg g^−1^) is the equilibrium adsorption amount at heavy metals equilibrium concentration *C_e_* (mg L^−−1^), where *K_f_* is Freundlich coefficient characteristic of the adsorption affinity of the adsorbent, and *n* is the linearity index. The validity of isotherm models used in this study is assessed by correlation coefficient (R^2^). The fitted results of all isotherm models were investigated in this study are presented in the Table S1. The results showed that Langmuir model with R^2^ higher than 0.99 fitted better than Freundlich model, indicating that Pb(II) and Cd(II) adsorption on MMSP-GO can be considered to be a monolayer adsorption process. The abundant oxygen-containing functional groups on the surface of graphene oxide made the adjacent oxygen atoms available to bind metal ions [27]. At the same time, the amino groups of MMSP had a strong affinity towards metal ions, and the possible adsorption mechanism could be reasoned out by the coordinate interactions. [33].

### 3.4. Adsorption of Pb(II)/Cd(II) by MMSP-GO Influenced by HA

**Figure 9:**
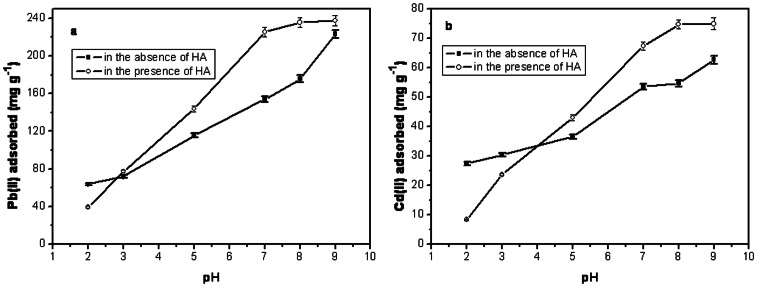
Effects of HA on the adsorption of heavy metal ions by the composites. Effects of HA on the adsorption of (a) Pb(II) and (b) Cd(II) on polyethylenimine-modified magnetic mesoporous silica and graphene oxide (MMSP-GO) composites.

We also investigated the influence of HA at an initial concentration of 10 mg L^−−1^ on the adsorption of heavy metal ions onto MMSP-GO composites within a pH range of 2.0 to 9.0. For both types of metal ions of Pb(II) and Cd(II), the adsorption capacity of the ion in the presence of HA was greater than in the absence of HA for MMSP-GO composites after a certain pH value (Figure 9). The two curves crossed at about pH 3.0 for Pb(II) and about pH 4.0 for Cd(II). Under pH neutral conditions, about 72 mg g^−1^ more Pb(II) was adsorbed in the presence of HA than in its absence, and about 14 mg g^−1^ more Cd(II) was adsorbed. The amounts HA adsorbed by MMSP-GO composites in the presence of Pb(II) or Cd(II) were about 83 and 87 mg g^−1^, respectively, under neutral conditions. The adsorption capacity of HA molecules remained roughly the same no matter which heavy metal ions were present. This implied that there was a specific site for HA adsorption on the MMSP-GO and that this site was not influenced by metal ion adsorption. We reported previously that PEI-modified MMSP microspheres could adsorb HA molecules efficiently across a wide pH range. [28] Nanoparticles coated with HA showed enhanced adsorption of heavy metal ions, such as Pb(II).[34]–[37] As with GO nanosheets, at pH>7.0, adsorption of Cd(II)/Pb(II) was hindered by the presence of 10 mg L^−1^ HA in the solution because the HA would bind to the adsorbent, competing with the metal ions. [27] On the contrary, the enhanced adsorption of the metal ions onto MMSP-GO composites in the presence of HA molecules may be attributed to the joint effects of the PEI-modified MMSP microspheres and the GO sheet. When HA is not present during adsorption, the metal ions may have been mainly adsorbed onto GO nanosheets, accompanied by only some adsorption onto MMSP. In contrast, in the presence of HA, most of the HA was trapped by the MMSP microspheres, and so metal ions were able to interact with the HA on the composite surface. Moreover, adsorption of HA molecules could neutralize the positive surface charge of the MMSP and enhance adsorption of heavy metal ions. In this way, in addition to the adsorption of metal ions onto the GO surface, extra metal ions could be adsorbed onto MMSP microspheres mediated by HA on their surfaces. We examined the sorption of HA by the composites through FTIR measurements. Description of the major transmittance bands in FTIR spectra of HA, MMSP-GO composites, MMSP-GO/HA/Pb^2+^ and MMSP-GO/HA/Cd^2+^ complex are presented in Figure S2. By comparison with bulk HA and pure MMSP-GO composites, the strengthened peak intensity and occurrence of new peak of bands at 1580, 2700 and 3900 cm^−1^ diminished markedly for both MMSP-GO/HA/Pb^2+^ and MMSP-GO/HA/Cd^2+^, which could result from the strong interactions of COOH, Phenolic OH, and OH of aliphatic alcohol with MMSP-GO composites. This result proved successful adsorption of HA by the composites.

### Conclusions

A facile and reproducible method was developed and used to prepare magnetic mesoporous silica-graphene oxide composites (MMSP-GO) with hierarchical structures and unique properties. These composites were suitable for synergistic adsorption of heavy metal ions and humic acid molecules. The magnetic mesoporous silica microspheres were synthesized and functionalized with PEI molecules, which provided abundant amine groups for chemical conjugation with the carboxyl group on GO sheets and enhanced affinity between pollutants and the mesoporous silica. Adsorption results recorded under different conditions indicated that this experiment was sufficient to prove that this system was capable of simultaneous removal of heavy metal ion and humic acid using MMSP-GO composites as the adsorbent. The underlying mechanism of synergistic adsorption of heavy metal ions and humic acid were discussed. This kind of composite adsorbent was able ot make use of both the large specific surface area and surface functionalities of GO and mesoporous structures. This allowed them to efficiently adsorb the heavy metal ions and HA, and the adsorption of HA onto the composites enhanced the adsorption efficiency of Pb(II) and Cd(II).

## Supporting Information

Figure S1 a. Effects of pH on adsorption of Pb(II) by MMSP and GO (Initial concentrations: 20 mg L^−1^; adsorbent loading: 100 mg L^−1^); b. Effects of pH on adsorption of Cd(II) by MMSP and GO (Initial concentrations: 20 mg L^−1^; adsorbent loading: 100 mg L^−1^).
(TIF)

Figure S2 FTIR spectra of bulk HA (a), MMSP-GO composites (b), MMSP-GO/HA/Pb(II) complex (c), and MMSP-GO/HA/Cd(II) complex (d).
(TIF)

Table S1 Constants and correlation coefficients of Pb(II) and Cd(II) adsorption by langmuir and Freundlich model.
(DOC)
