# Supplementary figures and images for: Synergistic Removal of Pb(II), Cd(II) and Humic Acid by Fe3O4@Mesoporous Silica-Graphene Oxide Composites
Source: PLoS One. 2013 Jun 11;8(6):e65634. doi: 10.1371/journal.pone.0065634 (PMC3679167; doi:10.1371/journal.pone.0065634)

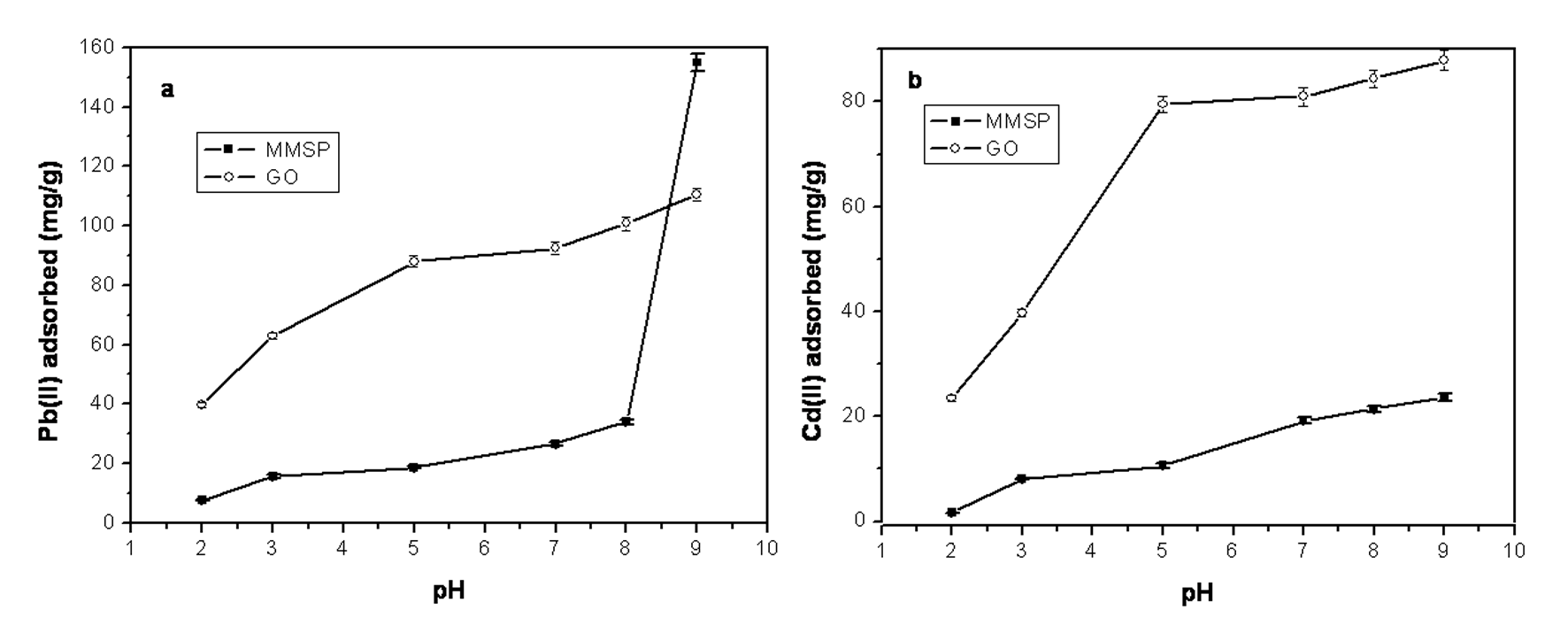

Supplement: Figure S1 — a. Effects of pH on adsorption of Pb(II) by MMSP and GO (Initial concentrations: 20 mg L−1; adsorbent loading: 100 mg L−1); b. Effects of pH on adsorption of Cd(II) by MMSP and GO (Initial concentrations: 20 mg L−1; adsorbent loading: 100 mg L−1). (TIF) [file pone.0065634.s001.tif]
