# Supplementary material for: Synergistic Removal of Pb(II), Cd(II) and Humic Acid by Fe3O4@Mesoporous Silica-Graphene Oxide Composites
Source: PLoS One. 2013 Jun 11;8(6):e65634. doi: 10.1371/journal.pone.0065634 (PMC3679167; doi:10.1371/journal.pone.0065634)
Supplement: Table S1 — Constants and correlation coefficients of Pb(II) and Cd(II) adsorption by langmuir and Freundlich model. (DOC) [file pone.0065634.s003.doc]

Table S1. Constants and correlation coefficients of Pb(II) and Cd(II) adsorption by langmuir and Freundlich model

| Model | Pb(II) | | Cd(II) | | | | |
| --- | --- | --- | --- | --- | --- | --- | --- |
|  | Constants | | *R*^2^ Constants | | | | *R*^2^ |
| Langmuir | *q*_max_=333.33  (mg g^-1^) | *b* = 0.12  (L mg^-1^) | | 0.994 | *q*_max_=166.67  (mg g^-1^) | *b* = 0.025  (L mg^-1^) | 0.996 |
| Freundlich | *k*_f_ =99.7 | 1/*n* = 0.62 | | 0.974 | *k*_f_ = 13.80 | 1/*n*=0.461 | 0.984 |
